# Inferring Bacterial Infiltration in Primary Colorectal Tumors From Host Whole Genome Sequencing Data

**DOI:** 10.3389/fgene.2019.00213

**Published:** 2019-03-15

**Authors:** Man Guo, Er Xu, Dongmei Ai

**Affiliations:** ^1^School of Mathematics and Physics, University of Science and Technology Beijing, Beijing, China; ^2^Basic Experimental of Natural Science, University of Science and Technology Beijing, Beijing, China

**Keywords:** unmapped reads, tumor tissue, colorectal cancer, infiltrating bacteria, maximum likelihood estimation

## Abstract

Colorectal cancer is the third most common cancer worldwide with abysmal survival, thus requiring novel therapy strategies. Numerous studies have frequently observed infiltrating bacteria within the primary tumor tissues derived from patients. These studies have implicated the relative abundance of these bacteria as a contributing factor in tumor progression. Infiltrating bacteria are believed to be among the major drivers of tumorigenesis, progression, and metastasis and, hence, promising targets for new treatments. However, measuring their abundance directly remains challenging. One potential approach is to use the unmapped reads of host whole genome sequencing (hWGS) data, which previous studies have considered as contaminants and discarded. Here, we developed rigorous bioinformatics and statistical procedures to identify tumor-infiltrating bacteria associated with colorectal cancer from such whole genome sequencing data. Our approach used the reads of whole genome sequencing data of colon adenocarcinoma tissues not mapped to the human reference genome, including unmapped paired-end read pairs and single-end reads, the mates of which were mapped. We assembled the unmapped read pairs, remapped all those reads to the collection of human microbiome reference, and then computed their relative abundance of microbes by maximum likelihood (ML) estimation. We analyzed and compared the relative abundance and diversity of infiltrating bacteria between primary tumor tissues and associated normal blood samples. Our results showed that primary tumor tissues contained far more diverse total infiltrating bacteria than normal blood samples. The relative abundance of *Bacteroides fragilis*, *Bacteroides dorei*, and *Fusobacterium nucleatum* was significantly higher in primary colorectal tumors. These three bacteria were among the top ten microbes in the primary tumor tissues, yet were rarely found in normal blood samples. As a validation step, most of these bacteria were also closely associated with colorectal cancer in previous studies with alternative approaches. In summary, our approach provides a new analytic technique for investigating the infiltrating bacterial community within tumor tissues. Our novel cloud-based bioinformatics and statistical pipelines to analyze the infiltrating bacteria in colorectal tumors using the unmapped reads of whole genome sequences can be freely accessed from GitHub at https://github.com/gutmicrobes/UMIB.git.

## Introduction

Many microbes inhabit human tissues and bodily fluids, forming a close symbiotic relationship with the host. The types, quantities, distribution features, genomes, and pathogenic mechanisms of human microbes vary greatly (Campo-Moreno et al., 2018). Generally, the total number of microbes (approximately 100 trillion) found in the human body is 10 times more than the number of human cells, and the number of genes they encode is 100 times more than that by the human genome. Those microbes play an important role in human health by regulating our digestive, immune, respiratory, and nervous system, and their dis-symbiosis has been associated with various diseases (O’Hara and Shanahan, 2006), such as inflammatory bowel disease (Norman et al., 2015), Crohn’s disease (Li et al., 2012), viral hepatitis (Kostic et al., 2012), and colorectal cancer (Littlejohn et al., 2016).

Using metagenomics approaches, researchers have found that colorectal tumorigenesis is mediated by toxins produced and secreted by the infiltrating bacteria that colonize the intestinal surface and trigger tissue inflammation, inducing otherwise normal cells to emit atypical signaling molecules. The whole process leads to local inflammatory reaction and the infiltration of innate immune cells, events which, in turn, accelerate tumor development (Chung et al., 2018; Dejea et al., 2018). For example, DNA damage may be induced in host cells owing to prolonged exposure to these toxins, initiating tumorigenesis (Zhu, 2013). Bacteria and their products can also facilitate viral infection in host cells, thereby inducing cancer (Lax and Thomas, 2002; Almand et al., 2017).

While direct experimental measurement of infiltrating bacteria remains challenging, the unmapped reads derived from host primary tumor tissue through whole genome sequencing (hWGS) data could allow us to study the pathogenic process involving microbes in colorectal cancer with *in situ* advantage and no additional cost. In the past, unmapped reads were often overlooked; however, recent studies have proved that they contain crucial microbial information relevant to tumorigenesis (Mangul et al., 2018). Nonetheless, as a consequence of the extremely low abundance of microbial DNA in comparison to host DNA, such research requires the development of rigorous and robust bioinformatics and statistical procedures.

Our approach was built on a growing number of studies measuring microbes in the biopsies of cancer patients via the reanalysis of reads that were not mapped to the human reference genome. Zhang et al. (2015) used MegaBlast to remap the unmapped reads of whole genome sequences of 27 gastric mucosal biopsies to microbial reference genomes, and they verified a close association between *Helicobacter pylori* and gastric tumors. Tang and Larsson (2017) conducted high-throughput sequencing to analyze the RNA or DNA from tumor tissues of patients with cervical adenocarcinoma and lymphoma and remapped the unmapped reads to the complete viral reference database to successfully detect known oncogenic viruses, as well as identify new viral strains in those tumors. Loohuis et al. (2018) studied 192 blood transcriptome samples of schizophrenic patients, applied MetaPhlAn to analyze the bacteria using unmapped reads, and identified *Planctomycetes* and *Thermotogae phyla* closely associated with schizophrenia.

Evidence gathered from those studies has established the rationale for reanalyzing microbes using unmapped reads as a cost-effective approach to investigate the interaction between microbes and disease progression. Accordingly, we herein report a novel cloud-based bioinformatics and statistical pipelines to analyze the infiltrating bacteria in colorectal tumors using the unmapped reads of whole genome sequences. We used SAMtools to extract the unmapped reads, PANDAseq to perform quality control, followed by the assembly of paired-end reads, as well as the use of Burrows-Wheeler Aligner (BWA) for remapping to bacterial reference genomes, and Genome Relative Abundance using Mixture Model theory (GRAMMy) to estimate their relative abundance. By analyzing the obtained relative abundance and diversity, we identified differential infiltrating bacteria between primary tumor tissues and associated normal blood samples.

## Materials and Methods

**FIGURE 1:**
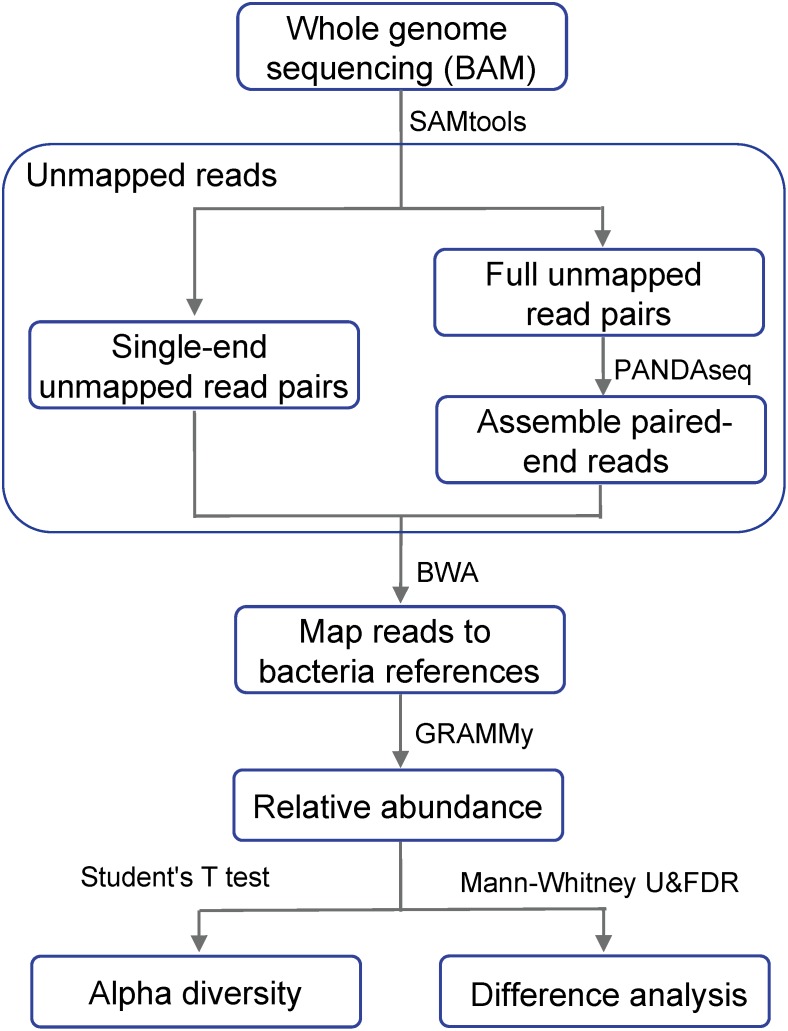
Flow chart showing the differential analysis of bacterial relative abundance using whole genome sequencing data. Whole genome sequencing BAM files are the result after mapping to the human reference genome.

Our data were downloaded from The Cancer Genome Atlas Colon Adenocarcinoma (TCGA-COAD) database, including the BAM-formatted whole genome sequencing data of 51 paired primary colon adenocarcinoma tumor and normal blood samples. Our bioinformatics pipeline was implemented using the Seven Bridge Cancer Genomics cloud platform, including four linked analytical components (SAMtools, PANDAseq, BWA, and GRAMMy) with their Docker images pushed up to the cloud platform. Figure 1 showed the flowchart of our approach for the analysis of differentially abundant bacteria using whole genome sequencing data. From the BAM files of the whole genome sequence data, we extracted reads that were not mapped to the human reference genome. Those reads were then mapped to a collection of human microbiome reference genomes to estimate the relative abundance of microbes.

### Extracting Unmapped Reads

We aimed to extract all unmapped reads, including both full read pairs (both ends of a read pair were unmapped) and single-end unmapped reads (one read end was mapped, while the other end was unmapped). Our bioinformatics procedures to extract such unmapped reads were as follows:

(a) *Full unmapped read pairs*. We first assembled the paired-ends sequencing reads and concatenated them into a longer single read to achieve more accurate alignment results. We extracted the full read pairs using the command “samtools view -u -f 4 -F264” and exported them as FASTQ files, followed by PANDAseq for assembling the paired reads. Since the initial output of the FASTQ files did not conform to the input format of PANDAseq, we wrote in-house script to add “/1” and “/2” to the ends of the IDs of the paired reads and then separated them into two FASTQ files per sample for the forward and reverse read, respectively. PANDAseq was then used to assemble the overlapping reads and filter out low-quality reads, setting its threshold as the default value of 0.6.
(b) *Single-end unmapped read pairs*. We extracted *single-end* unmapped read pairs with the command “samtools view -u -f 12 -F 256” and exported them as FASTA files.

The assembled *full unmapped read pairs* and the *single-end unmapped read pairs* were combined to obtain the complete set (FASTA files) of unmapped reads.

### Mapping and Calculating the Relative Abundance of Microbes

We used the Burrows-Wheeler Alignment tool (BWA) to remap the complete set of unmapped reads obtained in the previous step to the a collection of human microbial genome references. Our reference collection was downloaded from the NCBI human microbiome database: ftp://ftp.ncbi.nlm.nih.gov/genomes/HUMAN_MICROBIOM/Bacteria. Those reference genomes were sequenced, quality controlled and assembled by the Human Microbiology Program (HMP) (Methé et al., 2012) consortium. This reference collection contains 161 bacterial genus and it is also 519 of the most important bacterial species in the human body, including more than 900 strains. The reference collection was pushed up to the Seven Bridges Cancer Genomics cloud platform using the Cancer Genomics Cloud Uploader.

Next, we used GRAMMy (Xia et al., 2011), a mixture modeling and expectation- maximization algorithm-based maximum likelihood (ML) estimation tool, to determine the relative abundance of microbes. The tool overcomes the ambiguity of mapping to different microbial reference sequences that occur as a result of short read sequencing and a closely related reference collection to estimate the relative abundance accurately.

### Quality Control Post-abundance Estimation

We eliminated samples presenting extremely low relative abundance of all bacteria, except for *Propionibacterium sp.* We suspected *Propionibacterium sp.* to be a major contaminating species in both normal blood samples and primary tumor tissues of colorectal cancer, averaged as 0.9313 and 0.7142, respectively. The relative abundance of *Propionibacterium sp.* in normal blood samples was, on average, higher than that in the primary colorectal cancer tissues.

Because the amount of *Propionibacterium sp.* in both tumor and normal samples was disproportionately large, we decided to exclude its relative abundance from all analyzed samples and renormalized relative abundance of other species. We also excluded 5 primary tumor tissue samples and 15 normal blood samples, the total unmapped reads counts of which were less than five, presenting extremely low relative abundance of infiltrating bacteria. Finally, our analysis was based on the remaining 46 primary tumor tissue samples and 36 normal blood samples. It is noteworthy that a recent study has shown that metabolites of *Propionibacterium freudenreichii* can kill colorectal cancer cells, implicating its use as a probiotic for the prevention and treatment of early colorectal cancer (Casanova et al., 2018).

## Results and Discussion

**FIGURE 2:**
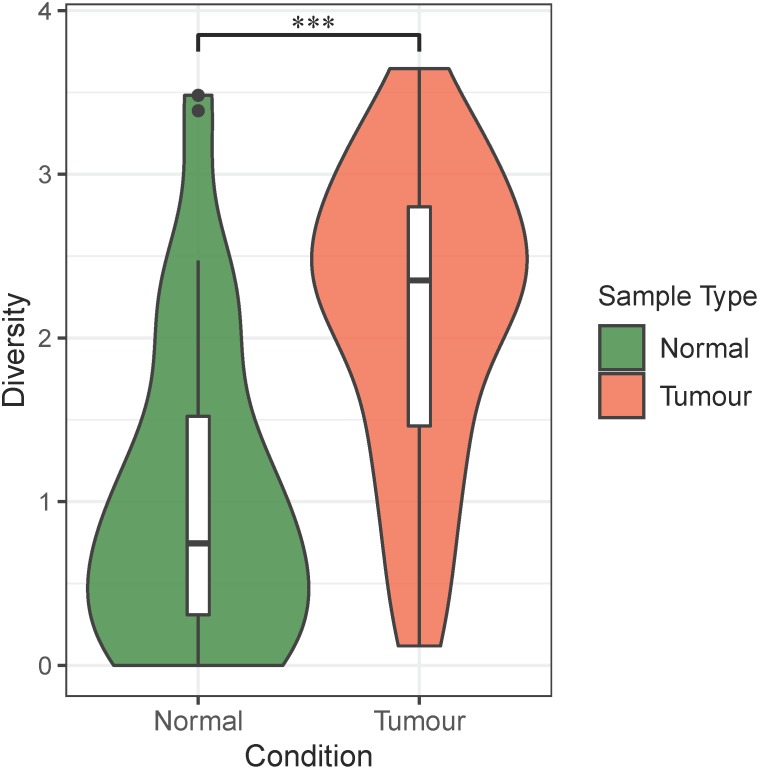
Alpha diversity of bacteria in the normal blood samples and primary tumor tissue samples. The violin plots show the alpha diversity of infiltrating bacteria in the normal blood and primary tumor tissue samples. The green color in the plot represents the normal blood samples, and the red color in the plot represents the primary tumor tissue samples. The “^∗∗∗^” symbol represents *P*-value < 0.001. Differential analysis was performed by Student’s *t*-test (*P* = 1.27E–06).

First, we calculated the Shannon’s indices of infiltrating bacteria for both normal blood samples and primary tumor tissue samples. As shown in Figure 2, the alpha diversity of bacterial communities indicated that the infiltrating bacteria in primary tumor tissues were significantly more diverse than those in normal blood samples. This finding was supported by previous studies, which showed that the alpha diversity of microbes in colorectal cancer biopsies was significantly higher than that in other samples, such as feces and saliva (Russo et al., 2018).

**FIGURE 3:**
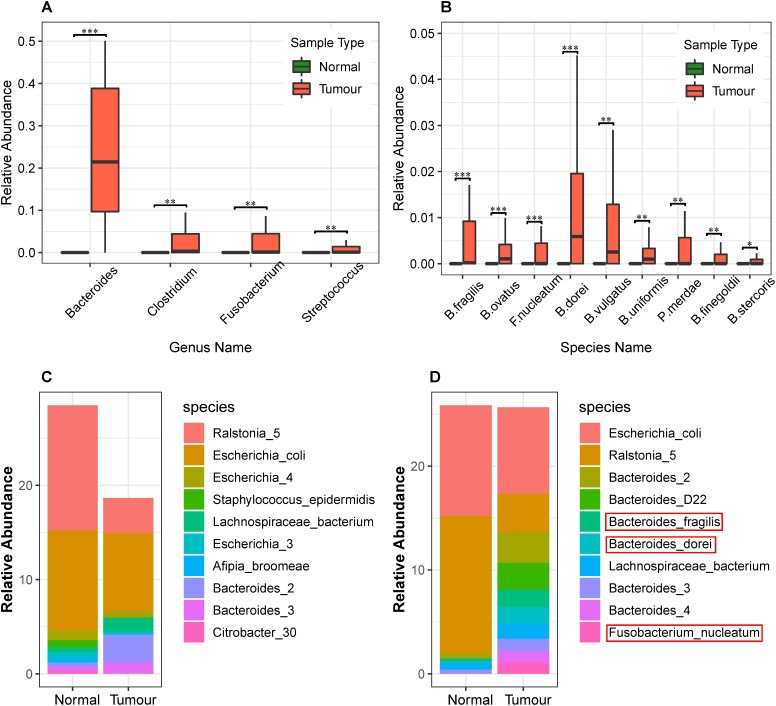
Differential analysis of the relative abundance of bacteria in the normal blood and primary tumor tissue samples. **(A)** Differential analysis of bacteria at the genus level in normal blood and primary tumor tissue samples. The Benjamini–Hochberg false discovery rate (FDR)-corrected non-parametric Mann–Whitney–Wilcoxon test was used to calculate the *P*-value and analyze the differences in bacteria. The box plots show bacteria significantly different at the genus level. The “^∗^” symbol represents *Q*-value < 0.05; the “^∗∗^” symbol represents *Q*-value < 0.01; and the “^∗∗∗^” symbol represents *Q*-value < 0.001. **(B)** Differential analysis of bacterial abundance at the species level in the normal blood and primary tumor tissue samples. To differentially analyze the diversity of bacterial species in the normal blood and primary tumor tissue samples, the Benjamini–Hochberg FDR-corrected non-parametric Mann–Whitney–Wilcoxon test was used. Letters B, F, and P in the *x*-axis represent *Bacteroides*, *Fusobacterium*, and *Parabacteroides*, respectively. **(C)** The stacked bar charts of the top 10 bacterial species enriched in the normal blood samples and their relative abundance in the primary tumor tissue samples. **(D)** The stacked bar charts of the top ten bacterial species enriched in the primary tumor tissue samples and their relative abundance in the normal blood samples.

Next, we identified the differential abundance of infiltrating bacteria between normal blood samples and primary tumor tissue samples. We used the wilcox.test() function in R software to perform a non-parametric Mann–Whitney–Wilcoxon test, followed by Benjamini–Hochberg procedure to compute the false discovery rate (FDR) and correct the obtained *P*-values. We identified the most significantly different genera (*Q*-value < 0.05), as shown in Table 1, and plotted them in Figure 3A. As we can see, *Bacteroides*, *Clostridium*, *Fusobacterium*, and *Streptococcus* were abundant in the infiltrated primary tumor tissues, but nearly absent in the normal blood samples.

**Table 1:** The most differentially abundant genera between tumor and normal samples (*Q*-value < 0.05).

Genus	*P*-value	*Q*-value (adjusted FDR)
Bacteroides	4.50E-09	4.81E-07
Clostridium	0.000377316	0.005046606
Fusobacterium	7.63E-05	0.002040633
Streptococcus	0.00095632	0.007309021


These findings were widely supported by previous literature. For instance, Flemer et al. (2017) showed that *Bacteroides spp.* in the mucosal microbiota of patients with colorectal cancer were more abundant compared to the normal control group. *Fusobacterium* recruits tumor-infiltrating immune cells to generate a pro-inflammatory microenvironment and promote tumorigenesis by triggering inflammation (McCoy et al., 2013). *Colitis* bacteria can alter host physiology to promote cancer. They disrupt the balance of intestinal microflora and introduce virulent genes that have been shown to promote tumor formation in mice (Walsh et al., 2014). In addition, many other species of *Clostridium* and *Streptococcus*, such as *Clostridium difficile* (Zheng et al., 2017), *Streptococcus gallolyticus* (Andres-Franch et al., 2017), and *Streptococcus infantarius* (Kaindi et al., 2018), were reported to be associated with colorectal cancer.

Whole genome sequence data allowed us to precisely identify the most abundant species. We identified such species and plotted the relative abundance of the top 10 most abundant species in stacked bar charts as shown in Figure 3C,D. As we can see, *Escherichia coli*, *Ralstonia spp.*, and *Bacteroides spp.* were abundant among all the primary tumor tissue samples and normal blood samples. Among these, *Ralstonia* was a common contaminant when DNA samples were screened (Salter et al., 2014), and its relative abundance may be a result of contamination. Both *E. coli* and *Bacteroides spp.* have important functional roles and are commonly found in the human body (Wexler, 2007).

In addition, we identified the most differentially abundant species between tumor and normal samples (*Q*-value < 0.05), as shown in Table 2, which included *B. fragilis*, *F. nucleatum*, *Parabacteroides merdae*, *B. dorei*, *B. vulgatus*, *B. stercoris*, *B. finegoldii*, *B. uniformis*, and *B. ovatus* (Figure 3B). It can be seen that the relative abundance of *Bacteroides fragilis*, *B. dorei*, and *Fusobacterium nucleatum* was also among the top 10 abundant species in the primary tumor tissue samples in this study, but they were much less abundant in the normal blood samples.

**Table 2:** The most differentially abundant species between tumor and normal samples (*Q*-value < 0.05).

Species	*P*-value	*Q*-value (adjusted FDR)
*B. fragilis*	1.36E-05	0.000715315
*B. ovatus*	1.31E-05	0.000715315
*F. nucleatum*	8.84E-06	0.000715315
*B. dorei*	1.88E-05	0.000850917
*B. vulgatus*	0.000112939	0.002974056
*B. uniformis*	0.000150549	0.00317157
*P. merdae*	0.000147964	0.00317157
*B. finegoldii*	0.000190032	0.003336119
*B. stercoris*	0.005728611	0.043100975


A subsequent literature search has validated these species as microbial markers of colorectal cancer. For instances, *B. fragilis*, also known as ETBF, secretes *B. fragilis* toxins (BFT) that induce immune cells to produce interleukin-17 (Wu et al., 2009). This lymphokine acts on intestinal mucosal cells to initiate the participation of more immune cells in the inflammatory response, thereby leading to the development of inflammation-related colorectal cancer (Kwong et al., 2018; Tilg et al., 2018). *F. nucleatum* adheres to and invades colonic epithelial cells, inducing tumor growth in patients with colorectal cancer (Bullman et al., 2017; Shang and Liu, 2018). In addition, *F. nucleatum* often presents in the human oral cavity to cause periodontitis, and it is reported to be a risk factor for colorectal cancer (Barton, 2017). Other identified bacterial species, such as *P. merdae*, *B. dorei*, and *B. vulgatus* (Cipe et al., 2015), are positively correlated with red meat intake and negatively correlated with the intake of fruits and vegetables (Feng et al., 2015). Red meat was widely recognized as a dietary factor linked to the development of colorectal cancer (Brenner et al., 2014). *B. finegoldii* and *B. dorei* can cause bacteremia (Lee et al., 2015), along with *B. Stercoris* (Lucas et al., 2017; Alomair et al., 2018), *B. uniformis*, and *B. ovatus* (Liang et al., 2014), and they were all reported to be correlated with colorectal cancer.

**FIGURE 4:**
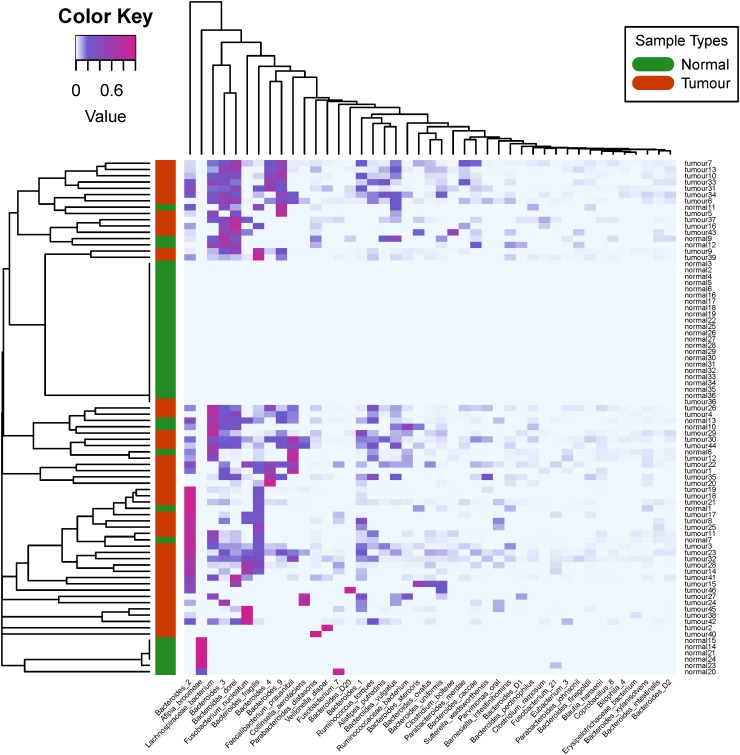
Heat map and biclustering analysis of different colorectal cancer tissue samples based on phylogenesis of the bacterial species. Forty-three different bacterial species among the 46 selected primary tumor tissue samples and 36 normal blood samples were used to prepare the heat map. The red color of the tree diagram on the left hand side represents the primary tumor tissue samples, and the green color represents the normal blood samples.

In Figure 4, we plotted the overall heat map of 43 bacterial species with significant differences. We used the R heatmap.2 function to draw the figure. The left side of the heat map demonstrates the clustering analysis of different samples using Spearman’s correlation coefficients between the relative abundance of bacteria. The figure clearly shows that the infiltrating bacteria of the primary tumor tissue sample were different from those of normal blood samples. The visible diversity of bacteria in the primary tumor tissue samples was significantly higher than that in the normal blood samples. This result is consistent with the findings from the differential analysis of alpha diversity. Interestingly, these 43 bacterial species only rarely present in most of the normal blood samples. In addition, the heatmap-based clustering analysis results showed that the primary tumor tissues of colorectal patients and normal blood samples were perfectly clustered with their sample types, revealing their distinct community structure.

## Conclusion

Cloud computing was developed recently in bioinformatics research (Zou et al., 2013; Guo et al., 2018). In this study, we developed a cloud-based bioinformatics pipeline to analyze unmapped reads from whole genome sequencing of human tumor tissues. The reads in the whole genome sequencing data not mapped to the human reference genome were extracted by SAMtools, followed by PANDAseq to assemble overlapping reads, BWA to remap them to the bacterial genome reference database, and GRAMMy to estimate relative abundance.

This pipeline was successfully applied to analyze the infiltrating bacteria of 51 pairs of primary colorectal cancer tumor tissue and normal blood samples. Group-based differential diversity and relative abundance analysis was used to identify microbial markers of colorectal tumor. Our results showed that the total infiltrating bacteria in primary tumor tissues was significantly more abundant than that observed in the normal blood samples. The relative abundance of such bacteria as *B. fragilis*, *B. dorei*, and *F. nucleatum* was significantly higher in primary tumor tissues as compared to normal blood samples. These bacteria are likely pathogenic microbial markers for colorectal cancer. A literature search validated our findings and revealed that these bacteria may induce tumor growth by adhering to and infecting the intestinal epithelial cells and secreting toxins.

## Data Availability

Publicly available datasets were analyzed in this study. This data can be found here: ftp://ftp.ncbi.nlm.nih.gov/genomes/HUMAN_MICROBIOM/Bacteria.

## Author Contributions

MG and DA conceived and designed the study and wrote the manuscript. MG and EX collected the datasets and created the workflow. MG and DA revised the manuscript. All authors read and approved the final manuscript.

## Conflict of Interest Statement

The authors declare that the research was conducted in the absence of any commercial or financial relationships that could be construed as a potential conflict of interest.
